# Scarcity amidst plenty: Regulating digital transformation

**DOI:** 10.3389/frma.2022.1004369

**Published:** 2022-11-30

**Authors:** Olufunmilayo B. Arewa

**Affiliations:** Temple University Beasley School of Law, Philadelphia, PA, United States

**Keywords:** digital transformation, digital divide, scarcity, inclusion, inequality, technology companies, Amazon, workers

## Abstract

Digital transformation has become a core aspect of lived experiences in recent years. Digital transformation has led to many aggregate benefits in the United States and throughout the world. The distribution of these benefits remains an issue of continuing contention. Digital transformation has occurred in contexts of significant disruption, both positive and negative. Although the positive aspects of disruption are often celebrated, potential negative consequences of digital transformation may not be adequately recognized. Digital transformation may, along with other factors, intensify existing societal divides, lead to greater inequality in many places, and contribute to a scarcity of opportunity for many people. Dealing with potentially adverse consequences of digital transformation requires flexible approaches to regulation and systematic use of metrics. Digital transformation also implicates policy issues, including those concerning technology infrastructure and education and training. Digital economy policies must take account of the requirements of an economy permeated with the effects of digital transformation. Addressing digital economy adversities will require greater attention to digital economy participation and inclusion. Fostering digital economy inclusion requires attention to both the distribution of digital economy benefits and preconditions for digital economy participation.

## Introduction

In November 2019 and again in 2021, workers across Europe and the U.S. went on strike against Amazon over wages and working conditions in Amazon warehouses. Some have identified these ongoing worker protests as reflecting a worldwide worker revolt against Amazon (Leon, 2021). As of early February 2022, Jeff Bezos, the founder and former CEO of Amazon, was the third wealthiest person in the world with an estimated fortune of some $187 billion,^1^ an increase of more than 60% since 2019, when his estimated wealth of $115 billion made him the wealthiest person in the world. Conditions for many Amazon workers have in many instances not been so favorable, which has led to protests and strikes.

Amazon is currently at the center of global debates about the role and responsibilities of corporations and how to best regulate digital economy technology companies and digital transformation more generally. Amazon highlights areas of uncertainty, change, and at times contestation driven by digital transformation. Digital transformation has fundamentally changed varied aspects of how businesses operate, which has had a broader aggregate societal impact globally. Digital transformation is complex and multifaceted and has been accompanied by changing societal, economic, and work conditions driven in part by technological innovation and broader digital economy trends.

The global activities of prominent technology companies, many of whom are based in the United States, have led to almost unparalleled plenty, in an era that has been likened to a new gilded age. At the same time, the activities of such firms highlight significant zones of inequality and scarcity of opportunity. Digital transformation draws attention to questions of sustainability and how digital economy economic growth may impact social, cultural, environmental, and other conditions in local and global contexts. Robust and sustainable economic growth with widely distributed opportunities will require focused policy attention to attenuating potentially detrimental aspects of the digital transformation that persist notwithstanding broader growth trends. This will require addressing digital economy trends that contribute to poor economic opportunities, particularly in regions and among groups and communities that have benefited to a lesser extent from digital era economic growth.

This paper will discuss potential benefits and negative consequences of digital economy trends in which digital transformation is a core aspect. It will also consider questions of inclusion as they relate to distribution of the benefits of the digital economy. In relation to questions of inclusion, this paper will consider people and locations that may not have experienced the benefits of the digital economy in any robust way. This paper will also discuss important prerequisites to enable digital economy participation (and thus likely foster inclusion) and implications of digital economy transformation for infrastructure, education, and training.

## Digital transformation, work, and discontent

Many call our current era the digital age, largely on account of the importance of technology and technological innovation as guiding forces in economic, business and sociocultural spheres (Johnson, 2006). The term digital economy describes fundamental changes in which digital transformation is a core aspect. This digital transformation has reshaped how we think about, share, and use knowledge and information (World Economic Forum, 2016). Webs of networked relationships have become widespread, often mediated by platform companies such as Amazon, Google, Facebook, WeChat, Instagram (owned by Facebook), TikTok, Twitter, Whatsapp (owned by Facebook), and Weibo.

Digital technologies and innovations are at the center of transformational changes that have led to a paradigm shift in business and society (Komarčević et al., 2017, p. 32). Trends reflecting automation and digitization have been evident in wide range of new technologies and new applications of existing technologies. A core aspect of digital transformation relates to data and information. Technologies today facilitate widespread dissemination of information, including visual images, and rapid communication to billions of people across the globe. As of April 2022, five billion people were active Internet users, with some 4.65 billion social media users (Statistica Research Department, 2022).

In business, digital transformation has involved a number of core principles, including flexibility, evident in dynamic networked business processes, decreased execution time, greater ability to customize, increased efficiency of processes and services due to the ability to evaluate data on a large scale, and more adaptable organizational structures (Schwab, 2016; Schwertner, 2021). Schwab of the World Economic Forum has highlighted the potential for technology to lead to “a supply-side miracle, with long-term gains in efficiency and productivity. Transportation and communication costs will drop, logistics and global supply chains will become more effective, and the cost of trade will diminish, all of which will open new markets and drive economic growth” (Schwab, 2016).

The digital economy also highlights the increasing business importance and value of information and other intangibles for many companies. This in turn underscores a shift in dominant business production and operation models to ones involving significant utilization of intangibles. Intangibles have contributed to a marked yet little studied transformation in business practices and sources of economic value for many firms. This transformation is only likely to intensify in an era of big data solutions. The COVID-19 pandemic has accelerated automation and digitization trends leading some to refer to the pandemic as the Great Digital Accelerator (Qureshi and Woo, 2022, p. 2).

Digital transformation has changed not only business, but society more generally. This digital transformation highlights the “flow from the exponential, digital, and combinatorial nature of progress with digital technologies... [that] is enriching our world and our lives more quickly than [previously thought, making this progress] the best economic news on the planet” (Brynjolfsson and McAfee, 2014). Digital transformation has the potential to raise incomes and improve the quality of life for many. However, those who have gained most from digital technologies may be consumers most “able to afford and access the digital world” (Schwab, 2016). For others not so able to afford and access digital worlds, digital transformation may lead to diminished quality of life, which draws attention to significant challenges present in digital economy contexts.

Digital transformation underscores broader economic trends. Technology is a key factor in long-term economic growth (Qureshi and Woo, 2022, p. 3).^2^^,^^3^ Notably, as digital technologies have boomed, productivity growth has decreased and economic growth has trended lower.^4^ Further, greater income inequality has come with the digital technology transformation: “income inequality has risen in all major advanced economies since the 1980s, and quite appreciably in several of them, [with] a particularly sharp increase in income concentration at the top end of the distribution” (Qureshi and Woo, 2022, p. 3)

The potential for digital transformation to disrupt labor markets is a factor, together with other complex elements,^5^ in increased digital economy inequality. As economists Brynjolfsson and McAfee have noted, digital transformation has presented significant work and wage challenges, which reinforce:

the idea... that as technology races ahead it's leaving some people behind. They want to work, to offer their labor to the economy, but their capacity as workers doesn't match the new environment. Technological progress is certainly not the only factor affecting jobs and wages—others include globalization and demographics—but we continue to believe that it's a major one. (Brynjolfsson and McAfee, 2014, p. xiii)

Automation is likely increasing labor market disruption: “[a]s automation substitutes for labor across the entire economy, the net displacement of workers by machines might exacerbate the gap between returns to capital and returns to labor. On the other hand, it is also possible that the displacement of workers by technology will, in aggregate, result in a net increase in safe and rewarding jobs” (Schwab, 2016). Schwab (2016) suggests that the impact on workers makes inequality a key economic concern and the “greatest societal concern” associated with digital transformation. The impact of digital transformation on workers has led to discontent and a pervasive sense of dissatisfaction and unfairness in a winner-takes-all economy that is a “recipe for democratic malaise and dereliction” (Schwab, 2016).

Notably, discontent may be exacerbated by:

the pervasiveness of digital technologies and the dynamics of information sharing typified by social media... In an ideal world, these interactions would provide an opportunity for cross-cultural understanding and cohesion. However, they can also create and propagate unrealistic expectations as to what constitutes success for an individual or a group, as well as offer opportunities for extreme ideas and ideologies to spread (Schwab, 2016).

The rapid pace of digital economy technology innovation also presents regulatory challenges. Legal and policy mismatch is a pervasive global digital economy concern. This mismatch has been evident in uncertainty about how to apply existing laws and regulations. Although the aggregate benefits of the digital economy are notable, the inequality exacerbated by digital transformations highlights potentially negative consequences of digital transformation that must be addressed from a policy perspective. The digital economy raises two key interrelated issues of inclusion and participation.

## The impact of digital transformation

Amazon exemplifies the benefits of digital transformation as well as sources of digital economy discontent. Amazon has been at the center of digital transformation for almost three decades. Amazon first emerged as a company that envisaged “new business models... to sell books according to novel modes” (Resca and Spagnoletti, 2014, p. 175). Amazon's website was thus a platform for its broader strategic framework, which at first linked internet users as customers to merchants and Amazon partners. Amazon's customers could also be sellers, which highlights Amazon's website as “an infrastructure for the mobilization of a large number of actors,” enabling transformation of customers into suppliers and competitors into partners (Resca and Spagnoletti, 2014).

Amazon's evolution has reflected deployment of both a platform metaphor and infrastructure metaphor to facilitate the construction of communities realized through varied digital technologies (Resca and Spagnoletti, 2014). Amazon expanded over time to broader aspects of logistics at different levels, including in becoming a provider of electronic devices and publisher of content (Resca and Spagnoletti, 2014, p. 176). In 2004, Amazon launched a cloud infrastructure service, Amazon Web Services (AWS), which continues to be the most successful cloud infrastructure provider globally (Miller, 2016). Cloud services such as AWS are often presented as essential tools for digital transformation.^6^

Amazon thus began as a company using technology to transform itself and its markets and later added business segments that enabled it to come an essential provider of technologies to facilitate digital transformation more generally. The growth of Amazon has led to enormous wealth for Amazon senior executives, including former CEO Jeff Bezos. The scale of Amazon's business makes Amazon working conditions of Amazon employees of considerable global interest. In 2021, Amazon was said to employ more than 1.6 million people full-time and part-time worldwide (Coppola, 2022). The relative distribution of the benefits of Amazon's digital transformation has drawn attention. In December 2020, one comparison noted that Jeff Bezos could give $105,000 to a large number of Amazon employees and still have wealth equal to his pre-COVID-19 wealth (Goodwin, 2020). Amazon highlights relative outcomes for many workers that have led to widespread protests. Disputes at Amazon also underscore the presence of scarcity of opportunity in the midst of plenty, at least for some.

Protests against Amazon have been global (Segal, 2021), with Amazon workers in 20 countries protesting and striking on Black Friday in November 2021 (Biron, 2021). Amazon and many other prominent digital economy companies have many workers who are not categorized as employees but rather as contractors, which is an issue of contention at a number of prominent companies (Parmeter, 2016). Wages, work conditions, and Amazon's treatment of unionizing activities have been a focus of protests (Biron, 2021). Worker compensation has been a key element of protests against Amazon, particularly for workers at Amazon warehouses. The scale of Amazon's operations as the largest online retailer in the world heightens the impact of Amazon's work environment. In the United States, for example, Amazon is the second largest private employer in the country; Amazon's facilities influence inflation, job markets, and labor standards (Herrera, 2021). Amazon distribution center activities may have a downward impact on wages. An analysis by *The Economist* suggests that:

[f]lat or falling industry wages are common in the cities and towns where Amazon opens distribution centers... Government figures show that after Amazon opens a storage depot, local wages for warehouse workers fall by an average of 3%. In places where Amazon operates, such workers earn about 10% less than similar workers employed elsewhere.^7^

The activities of Amazon and other companies at the forefront of digital transformation are of particular concern because of the public and private costs that such companies may impose. Amazon plays an important role in job creation, including in the United States (Saxena, 2021). Although Amazon claims that its benefits are “industry-leading” available evidence suggests that this is not in fact the case (Saxena, 2021). As was evident in 2018 during a visible and public competition among U.S. states for a second Amazon headquarters (HQ2), Amazon receives significant public subsidies:

Amid the guessing game, the company got information from dozens of cities about how much they would pay for a strong Amazon presence, valuable data that it will no doubt use to expand. (Streitfeld, 2018).

A 2022 UNI Global Union and Good Jobs First Report estimates that Amazon has received more than $4.18 billion in economic development subsidies in the United States and at least $4.7 billion in subsidies worldwide (Thomas et al., 2022). Low wages at Amazon and other companies also impose costs on taxpayers because of reliance by workers on federal and state benefits. Use of federal and state benefits by low-wage workers was estimated in 2015 to cost $152.8 billion per year (Jacobs et al., 2015, p. 2). In Arizona, a third of Amazon employees were said to rely on food stamps in 2017, with Amazon having received some $4 million in subsidies from Arizona (Brown, 2018). In 2015, 56% of combined state and federal spending on public assistance went to working families (Jacobs et al., 2015, p. 2).

Amazon workers have protested about more than their compensation and benefits. Protests at Amazon also relate to employment practices and general working conditions (Sainato, 2020), some of which reflect important consequences of digital transformation for many workers. Of particular concern are technologies that enable extensive monitoring and surveillance of employees by their employers (Williams, 2021; Klippenstein, 2022). The impact of automated human resources applications and employee surveillance technologies on Amazon's large workforce have also been noted to be issues of concern for Amazon workers (Greene, 2021; Kantor et al., 2021). A number of Amazon warehouses have attempted to form unions, resulting in ongoing contestation between Amazon and its employees over unionization efforts. One warehouse in Staten Island, New York, voted to form Amazon's first union in early 2022 (Weise and Scheiber, 2022). This union vote was characterized as a rebuke of Amazon's treatment of its employees (Weise and Scheiber, 2022).

As the successes of companies such as Amazon illustrate, the distribution of digital economy benefits is a fundamental issue of concern for our era of digital transformation. Digital transformation has had many spillover effects that have benefitted many people. Spillover effects from innovative technologies have been an important feature of digital era economic growth. Digital economy growth has often been centered in specific geographic clusters, of which Silicon Valley has to date been the most prominent. The effective diffusion of technology and creation of spillover effects are often noted as core aspects of successful digital economy geographic clusters.^8^

Despite variations in stock market values, particularly after a significant decline in valuations in early 2022, technology companies today are enormously powerful. The dominance of technology companies has broader economic implications. Notably, the information sector is “particularly consolidated, with nearly three-fifths of its output squeezed into just a few dozen [U.S.] counties” (Tartar and Pickert, 2019). Growth in the United States in increasingly concentrated in just 1% of counties; these 31 counties accounted for 32.3% of United States gross domestic product in 2018 (Tartar and Pickert, 2019). While these counties made up over 32% of US GDP, they only contained 26.1% of employed people and 21.9% of the population (Tartar and Pickert, 2019). This increased geographic concentration is evident in urban areas and around the coasts, and all 31 counties included or were near major cities (Tartar and Pickert, 2019). Although other sectors, including finance and the arts, are highly concentrated, the concentration and dominance of the information sector have implications for patterns of inequality.

For many people, adversity has accompanied digital transformation. Reducing the adverse effects of digital transformation while maintaining the benefits of transformation should be a core digital economy policy focus. Higher levels of income inequality are an important potential consequence of digital era adversity globally. The costs of digitization are not evenly distributed: “[d]igitization contributes to more inequality both through job displacement that leads to changes in the distribution of earnings in favor of higher skills, and through a drop in the labor income share in sectors most exposed to automation as well as in the whole economy” (Bourguignon, 2022, p. 179).

Digital transformation thus generates costs that reduce overall digital economy benefits. Costs associated with digital adversities may even reduce overall economic benefits of digital transformation. For example, rising inequality reduces growth and aggregate demand because higher-income households that now receive a higher share of income have greater luxury to save money. The Economic Policy Institute estimates that the rise in inequality in the United States since the late 1970s has reduced aggregate demand by some 1.5% of GDP annually (Bivens and Banerjee, 2022, p. 3).

The gap between the poor and the super-rich is readily apparent in Silicon Valley, where the “homeless are the most visible signs of poverty in the region” (Rotman, 2014). In 2013, Silicon Valley median income was $94,000, well above the national median of some $53,000. At this same time, some 31% of jobs in Silicon Valley paid $16 per hour or less (Rotman, 2014), which is well-below what would be needed to support a family in Silicon Valley. At that time, the poverty rate in Santa Clara County, in the core of Silicon Valley, was some 19% (Rotman, 2014). In the years following the 2008 financial crisis, Silicon Valley experienced significant growth yet widening inequality. The 2020 Silicon Valley Index notes:

Income inequality in Silicon Valley is at a historic high, and 13 percent of households hold more than 75 percent of the region's wealth. Though per capita income and average wages continue their upward trend, rising median household income reflects the shifting distribution of households into the higher income ranges. Thirteen percent of the region's households have more than $1 million in net assets, while 37 percent have less than $25,000 in savings.^9^

In the San Francisco Bay Area more generally, technological innovations have created immense wealth for some but have also contributed to greater socioeconomic inequality. Debates surrounding the cost of housing, the placement of bus stops that carry workers from San Francisco to Silicon Valley, dealing with human waste from the large number of homeless people on the streets of San Francisco, and other social concerns highlight points of tension that have emerged in the midst of immense wealth and prosperity, at least for some.

Although levels of inequality in the developed world are higher in the United States than almost any other developed country (Siripurapu, 2022), significant divergences in income distribution are present in other developed countries (Piketty, 2017). In Britain and France, for example, accumulated wealth was noted in 2014 to be “returning to relative levels not seen since the First World War” (Rotman, 2014). COVID-19 magnifies these existing trends because, as noted by the Secretary-General of the United Nations, the digital divide has become a matter of life and death (United Nations, 2020).

The dominance of technology companies, including those that emerged in Silicon Valley and other technology clusters, underscores the highly concentrated nature of the digital economy activity in much of the developed world. The IMF notes that regional disparities in the “average advanced economy have risen since the late 1980s, reflecting gains from economic concentration in some regions and relative stagnation in others” (International Monetary Fund, 2019). These gaps have significant implications for people living in lagging regions, including poorer health outcomes, lower labor productivity and longer times in adjusting to trade shocks (International Monetary Fund, 2019). Even within urban areas that have experienced gains from economic concentration, such gains may not be distributed evenly among all communities within such areas. This unevenness means that even areas that are not lagging by aggregate statistics may have members of the community that experience circumstances like those in lagging regions. In the United States, for example, immigrant households experience a significant digital divide and lack access to tools such as computers and smartphones (Cherewka, 2020).

### The digital economy and scarcity

In an era of digital transformation, scarcity amidst plenty is a key element of lived experiences for many people. For many, even prior to the advent of COVID-19, real and robust economic and other opportunities appeared increasingly scarce, contributing to a scarcity of opportunity that has been particularly evident in varied contexts involving countries, regions, industries, and communities (Arewa, 2018). The perception and reality of scarce opportunities reflects policy failures to address diminishing opportunities for social mobility and advancement in varied parts of the world (Semuels, 2016; Alderman, 2019, p. B1; Kimmelman, 2018, p. A4). Opportunities for social and economic mobility and the regulation of new technologies and services have become critical policy issues globally and touch upon the removal of sources of unfreedoms identified by economist Amartya Sen (2000).

Scarcity of opportunity may be apparent in a range of areas, including lack of security or access to education (including as a result of existing education funding models), food, affordable housing, and healthcare and other essentials, labor market disruption, lack of available opportunities for one's children, low wages, low growth, lack of retirement security, high levels of indebtedness, including from loans taken out to finance education, limited access to finance on non-exploitative terms, and inadequate access transportation and other infrastructure (Arewa, 2018, p. 1031–1033). In the United States, scarcity of opportunity has contributed to increasing economic and financial instability for more than two-thirds of Americans in the years leading up to the COVID-19 pandemic (Newkirk II, 2019; Andres and Shaw, 2020).^10^ This pattern is evident in other parts of the world.^11^ COVID-19 has also drawn attention to inadequacies in essential digital economy infrastructures.^12^

## Fostering digital economy inclusion

Fostering digital economy inclusion requires attention to both the distribution of digital economy benefits and the preconditions for digital economy participation. In the digital economy, lack of access to broadband and other characteristics of digital divides contribute to poor economic opportunities and may also reflect systematic social deprivation, scarcity, and neglect of public facilities.

### Digital economy participation

Full access to digital economy opportunities requires access to tools that facilitate digital participation, including broadband and devices to access the Internet. Lack of access to essential digital economy tools is a global problem. The digital divide is a global issue of concern that may manifest in different ways in varied contexts.

In the United States, a February 2020 study suggested that the Federal Communications Commission (FCC) underestimated the number of Americans that lack access to broadband (Busby and Tanberk, 2020). According to FCC data, at the end of 2017, 21.3 million Americans lacked access to high-speed broad band (defined using the FCC benchmark of at least 25/3 Mbps) (Federal Communications Commission, 2019, p. 2). BroadbandNow checked the FCC's data and estimated that 42 million Americans did not have access to wired or fixed wireless broadband (Busby and Tanberk, 2020). The FCC undercounting of broadband access tends to be greater in states with higher rural populations. The lack of access to broadband exacerbates gaps, particularly in rural areas and among other communities where many may already be left behind (Tramontano, 2017; Hendrickson et al., 2018, p. 12; Wuthnow, 2018). Questions about variations in economic outcomes and rural digital economy infrastructures are not limited to the United States. In Germany prior to the COVID-19 pandemic, for example, Internet speeds in rural areas were slower: “[a]t the moment, Germany's rural areas are still leagues away from their urban counterparts when it comes to internet access. Only 75.1 percent of rural areas achieve 30 Mbit/s internet speed whereas cities are at 97.4 percent according to official government numbers” (Franz, 2020).

## The COVID-19 great digital accelerator and digital divides

The COVID-19 pandemic highlights core features of the differential impact of the digital economy, as well as gaps evident in the digital divide and other important digital economy measures. For example, the United States has experienced a two-track COVID-19 recovery in which some workers, companies, and regions emerged from the COVID-19 driven economic contraction “fine or even stronger,” while others remained “mired in a deep decline with an uncertain path ahead” (Morath et al., 2020). This recovery was said to be shaped like the letter K, with “well-educated and well-off people, businesses tied to the digital economy or supplying domestic necessities, and regions such as tech-forward Western cities... prospering [with] lower-wage workers with fewer credentials, old-line businesses and regions tied to tourism and public gatherings” on the bottom arm of the K (Morath et al., 2020). The COVID-19 pandemic thus magnified existing digital economy trends and gaps among countries, regions, industries, and workers.

Discussion of the digital divide in the United States is not new but continues to highlight ways in which access to and uses of technology may be unevenly distributed (Wyatt et al., 2000; Wilhelm, 2004; Henwood and Wyatt, 2019). The digital divide relates to the “growing gap between those with access to telephones, modems, computers, and the Internet, and those without such access: the information rich versus the information-poor” (Leggon, 2006). Information wealth and information poverty likely track wealth and poverty more generally, at least to some extent. As a result, the digital divide has consequences that extend far beyond the digital world. As Julie Cohen has noted:

A ‘digital divide' is never only digital; its consequences play out wherever political and economic decisions are made and wherever their results are felt... In addition, it is equally important to consider how a digital divide might alter other resource distributions that inhere in social space. If the haves increasingly shop online while the have-nots shop in ‘real space,' the real-space distribution of goods, services, and employment patterns likely will change, and with it the real-space distribution of all of the activities that make up the commerce of daily life (Cohen, 2007).

Many aspects of digital technologies enhance lives. Other aspects of the digital economy may be troubling. The benefits of digital era technologies and their spillover effects are not evenly distributed, which has significant implications for development both among and within countries. For example, in the United States, even without the uneven geographic distribution of prominent digital economy activities, the digital era has unfolded in ways that may in some instances magnify existing inequalities.

## Regulating digital transformation

The presence of scarcity in the midst of plenty highlights the importance of regulating digital transformation. Digital transformation has posed significant challenges for existing legal and regulatory frameworks and in turn has serious implications for a broad range of people, including users of such firms' products and services and workers. The adoption of new technologies often leads to debates about how laws and regulations should apply to such technologies. These are essentially questions about legal and regulatory mismatch that might come with introduction of new technologies and new uses of existing technologies. Thus, mobile phones, mobile phone apps, Uber, and varied other technologies and services have required reassessment and varying degrees of reform of legal and regulatory approaches that might have been put in place well-before the advent of such technologies. This reassessment involves a broad range of areas, including laws and regulations relating to working conditions, privacy and security of personal information, intellectual property, and taxes, among others.

Amazon exemplifies some of the transformations that have come with the digital economy. These transformations create opportunities for entrepreneurs to build powerful companies with significant market power and, in many instances, generate large fortunes. However, significant dislocations may come with such transformations, including dislocations that impact legal and regulatory frameworks, and disruptions that impact the lives of employees (Wilson, 2019).

Jeff Bezos's wealth was accumulated in a world of significant insecurity for many Amazon workers. In addition, in a world of increasing wealth inequality and changing societal, economic, and work conditions driven in part by technological innovation and broader digital economy trends (Wilson, 2019), the potential uncertainties of employment in the “gig” economy is increasingly an issue for a broad range of workers.

The term “gig” comes out of musical performance contexts in which musicians performed short engagements or “gigs” (Graves, 2018). The employment circumstances of these musicians was often precarious. Well-before the digital economy, many performing musicians experienced work circumstances that reflect core digital “gig” economy issues (Torpey and Hogan, 2016). These issues include questions about employment status, which is a significant issue for ride-hailing services such as Uber and Lyft. Uber faces varied regulatory challenges and has fought regulatory battles all over the world, including in London, where a court in late September 2020 permitted Uber to renew its ride-hailing license for an 18 month period.^13^ Six days after Uber received its license renewal, its competitor Ola was not permitted to renew its ride-hailing license based on public safety concerns (Shead, 2020). Uber's past regulatory breaches were key points at issue in the Uber London license case.^14^ Uber has also in the past had a toxic internal culture.^15^

Uber has had a culture of rule breaking that is not uncommon today. Yglesias notes that Uber “gained initial traction in the marketplace thanks to a pirate-ship mentality that viewed willingness to break rules as a core competitive advantage” (Yglesias, 2017). This approach to legal and regulatory compliance in contexts of new technologies may present profound challenges for lawmakers, regulators, workers, and customers.

Digital economy companies may be difficult to regulate. The activities of such companies may also contribute to scarcity of opportunity. In addition to their impact on wages and work conditions, core business activities of digital economy and other companies may exacerbate conditions of scarcity. A continuing global debate exists, for example, about the impact of Airbnb on housing scarcity (Cox and Haar, 2020; Li et al., 2021). The scale of Airbnb, together with other digital economy trends, including Wall Street and investment fund activity in the housing sector, and vacant homes held off market,^16^ may reinforce housing scarcity trends (Brumer-Smith, 2022). Vacant homes are also potentially a reflection of a widening wealth gap (Branson, 2020), which highlights how varied trends may in aggregate reinforce patterns that exacerbate scarcity.

Notably, however, regulators may lack understanding, lack technological capacity, have insufficient regulatory capacity, or not have effective ability to regulate activities rooted in digital era business practices and cultural assumptions (of both companies and consumers). In addition to a rule-breaking approaches, digital economy companies have effectively leveraged their networked connectivity to consumers to undertake campaigns in opposition to attempts to regulate them. The success of digital economy companies, many of whom are profitable or at least very well-funded, has enabled such companies to accumulate resources that can make them formidable opponents of attempts to regulate them.

Airbnb, for example, has launched a “guerilla war” against local governments that have attempted to require Airbnb hosts to collect taxes and when such governments attempt to enforce zoning laws that might limit the number of Airbnb listings (Nieuwland and van Melik, 2020):

In the past five months alone, the company has spent more than half a million dollars to overturn regulations in San Diego and has sued Boston, Miami, and Palm Beach County over local ordinances that require Airbnb to collect taxes or remove illegal listings. Elsewhere, Airbnb has fought city officials over regulations aimed at preventing homes from being transformed into de facto hotels and requests from tax authorities for more specific data about hosts and visits.... Airbnb is engaged in “a city-by-city, block-by-block guerrilla war” against local governments, says Ulrik Binzer, CEO of Host Compliance, which helps cities draft and enforce rules for short-term rentals, sometimes putting it at odds with hosting platforms. “They need to essentially fight every one of these battles like it is the most important battle they have” (Martineau, 2019).

Uber and Lyft united in opposition to a 2019 California law that would have required them to hire workers as employees, not independent contractors. Assembly Bill No. 5 (AB-5) expanded the California Supreme Court decision in *Dynamex Operations West, Inc. v. Superior Court of Los Angeles*.^17^ AB-5 added Section 2750.3 to the California Labor Code,^18^ creating a “presumption that a worker who performs services for a hirer is an employee for purposes of claims for wages and benefits arising under wage orders issued by the Industrial Welfare Commission.”^19^

In response to AB-5, Uber, Lyft, and DoorDash, later joined by Instacart and Postmates, sponsored a ballot measure (Proposition 22). Proposition 22, which classified app-based workers as independent contractors who generally would not be covered under California labor laws (Mollaneda, 2021), was approved by California voters on November 3, 2020, with a vote of 58.6% in favor of the ballot measure (Ballotpedia, 2022). Gig economy companies wrote, sponsored, and funded a pro-Proposition 22 campaign, spending some $200 million in support of their efforts, making Proposition 22 the most expensive ballot initiative in California history (Mollaneda, 2021). Drivers and the Service Employees International Union then filed suit in Alameda County Superior Court, arguing that Proposition 22 was unconstitutional.

In August 2021, Proposition 22 was ruled unconstitutional in part because Section 7451 “limits the power of a future legislature to define app-based drivers as workers subject to workers' compensation law.”^20^ Gig economy companies indicated that they would appeal this ruling (Zaimes and Kreeger, 2021). Given the importance of Proposition 22 for gig economy business models and the implications of Proposition 22 for gig economy workers, this legal case is likely to be fiercely fought. Gig economy companies are fighting battles about the employment status of gig economy workers in multiple locations. In June 2022, gig economy companies supported a ballot measure in Massachusetts similar to Proposition 22 that would guarantee a minimum wage for workers but limit workers' access to benefits given employees (Browning, 2022). In December 2021, the European Commission introduced a draft directive that would give people working through digital platforms minimum wage and other protections (European Commission, 2021; Satariano and Peltier, 2021; Boesen and Pedersen, 2022).

The employment status of workers at Uber, Lyft, and other digital economy firms reflects uncertainties about employment status in relation to issues of control and other determinants of employment status that are not unique to such companies (Dubal, 2017), but which present greater challenges today due to scale and other factors. In a world of rising inequality, the work status of “gig” economy workers or people working through digital platforms may be precarious and may exacerbate insecurity. Although some workers may enjoy the flexibility of the gig economy, others may be forced to work for gig economy firms because other opportunities may be scarce or not be readily available to them.

## Policy approaches to digital economy transformation

Digital economy companies may disrupt more than markets. The scope of potential disruption may extend far beyond the areas within which such companies operate. Such companies have reflected and portend continuing changes in how we interact, work, play, live, and regulate. The activities of prominent digital economy technology companies are being increasingly scrutinized. Part of this scrutiny reflects renewed and likely sustained regulatory attention to such companies. In a post-COVID-19 world, this scrutiny must also take account of the broader societal impact of such companies, including in connection to available opportunities and questions related to inequality.

Digital economy inclusion will require assessment of varied policies, including in relation to regulation generally, taxation, and tools to facilitate digital era participation. The identification and development of metrics for measurement of digital era adversities should be a core aspect of targeted digital economy policies.

As a result of digital economy transformation, the economy today looks markedly different than it did even as recently as 20 years ago.^21^ Digital economy transformation requires innovative approaches to regulation in a complex arena of multiple and potentially overlapping areas. Regulation in digital economy contexts requires flexible and responsive regulatory approaches based on clearly understood objectives in varied contexts, including in accessing government services, in relation to workplace practices, and with respect to privacy and data security, to name three critical areas.

Regulation in the digital economy should be based on identifiable metrics with ongoing assessment of which policies meet such metrics and when existing metrics need reconsideration. Digital economy inclusion in the United States will also likely require attention to tax policy and varied infrastructures. Estimates suggest that the wealthiest in the United States and corporations pay lower tax rates than the average citizen (Leiserson and Yagan, 2021; Oxfam America, 2022).

Infrastructures that facilitate digital era participation are an additional area where policy interventions may be needed. In addition to infrastructures that enable networked connectivity, including Internet access, the digital era requires innovative approaches to education and training at all levels (Alenezi, 2021). For example, traditional approaches to K-12 education are “struggling to equip students with the skills in the most demand among the nation's leading businesses”^22^ (Brynjolfsson and McAfee, 2014, p. 208–209). A 2018 Report notes that “[m]any universities are developing specific digital strategies in reaction to the massive shift toward using new technology, yet lack the vision, capability or commitment to implement them effectively (see text footnote 22).” In addition to not sufficiently incorporating technology in higher education strategies, even before the pandemic, universities were not training students for digital economy participation: “[b]efore Covid, higher education was facing a crisis of employability as nearly half of all college students were graduating into underemployment. This crisis has been building for decades. While colleges have continued to do a reasonably good job of preparing students with the cognitive skills they need to become successful professionals... employers have changed” (Craig, 2021).

The COVID Great Digital Accelerator has highlighted significant gaps in adjusting to digital transformation. These gaps require flexible and focused policies as part of a broader regulatory and policy transformation to accompany digital economy changes that have already taken place as well as those yet to come. This regulatory and policy transformation must not only regulate digital transformation but must also itself make more effective use of digital technologies.

## Data availability statement

The original contributions presented in the study are included in the article/supplementary material, further inquiries can be directed to the corresponding author/s.

## Author contributions

The author confirms being the sole contributor of this work and has approved it for publication.

## Conflict of interest

The author declares that the research was conducted in the absence of any commercial or financial relationships that could be construed as a potential conflict of interest.

## Publisher's note

All claims expressed in this article are solely those of the authors and do not necessarily represent those of their affiliated organizations, or those of the publisher, the editors and the reviewers. Any product that may be evaluated in this article, or claim that may be made by its manufacturer, is not guaranteed or endorsed by the publisher.

## Footnotes

^1^The Real-Time Billionaire's List, Forbes. Available online at: https://www.forbes.com/real-time-billionaires/#36162e13d788.

^2^“Technological change—improvement in the instructions for mixing together raw materials—lies at the heart of economic growth” (Romer, 1990).

^3^Noting in Southern African context that certain technological innovation indicators have significant positive relationship with per capital economic growth in the long run (Anakpo and Oyenubi, 2022).

^4^Qureshi and Woo (2022), p. 3; see also Brynjolfsson and Petropoulos (2021).

^5^In addition to technological change, globalization, tax policies, reduced worker bargaining power, and racial and gender discrimination are factors in increased income inequality in the United States (Siripurapu, 2022).

^6^AWS. Public Sector Digital Transformation. Available online at: https://aws.amazon.com/government-education/digital-transformation/?public-sector-resources-dt.sort-by=item.additionalFields.sortDate&public-sector-resources-dt.sort-order=desc (Giemzo et al., 2020).

^7^*What Amazon Does to Wages*. The Economist (2018). Available online at: https://www.economist.com/united-states/2018/01/20/what-amazon-does-to-wages.

^8^Kenney and von Burg (1999); see also Frischmann and Lemley (2007).

^9^Joint Venture Silicon Valley, Institute for Regional Studies, 2020 Silicon Valley Index, at 20. Available online at: https://www.paloaltoonline.com/news/reports/1581544002.pdf.

^10^Financial Health Network, U.S. Financial Health Pulse 2019 Trends Report. Available online at: https://s3.amazonaws.com/cfsi-innovation-files-2018/wp-content/uploads/2019/12/16161507/2019-Pulse-Report-FINAL_1205.pdf.

^11^Although this article focuses on digital transformation in the United States, similar issues are relevant in many places. In Nigeria and other countries in Africa: “scarcity of opportunity is pervasive [and]... limits access for many to education, housing, electricity, clean water, healthcare, employment, economic possibilities, and other things.” (Arewa, 2021).

^12^For example, many developing countries lack local capacity to participate in important aspects of digital economy transformation. During the COVID-19 pandemic, capacity to produce vaccines became a key concern and exacerbated patterns of vaccine exclusion in which developed countries have hoarded vaccines produced in such countries. O. Arewa, Covid-19 Colonial Hangovers, Exclusion & Public Health in Africa.” Draft on file with author.

^13^In the Matter of an Appeal under the Private Hire Vehicles (London) Act 1998 between Uber London Limited (Appellant) and Transport for London (Respondent) and London Taxi Drivers' Association (Interested Party), In the Westminster Magistrate's Court, September 28, 2020. Available online at: https://www.judiciary.uk/wp-content/uploads/2020/09/Uber-v-TFL.pdf.

^14^Can Uber Overcome Its Regulatory Obstacles? Wharton Podcast, (2019). Available online at: https://knowledge.wharton.upenn.edu/article/can-uber-overcome-regulatory-obstacles/.

^15^Covington and Burling Recommendations. Available online at: https://drive.google.com/file/d/0B1s08BdVqCgrUVM4UHBpTGROLXM/view.

^16^*Wall Street's Housing Grab Continues*. *The Economist* (2022). Available online at: https://www.economist.com/finance-and-economics/2022/05/25/wall-streets-housing-grab-continues (Putzier, 2022).

^17^Dynamex Operations West, Inc. v. Superior Court, S222732, April 30, 2018. Available online at: https://cases.justia.com/california/supreme-court/2018-s222732.pdf?ts=1525107724.

^18^As Amends the Law on November 18, 2019. Available online at: https://leginfo.legislature.ca.gov/faces/billCompareClient.xhtml?bill_id=201920200AB5&showamends=false.

^19^AB-5 Legislative Counsel's Digest. Available online at: https://leginfo.legislature.ca.gov/faces/billTextClient.xhtml?bill_id=201920200AB5.

^20^Hector Castellanos, et al. v. State of California, et al., Case Number S266551 (Alameda County Superior Court), at 11. Available online at: https://42z27se827b1zpvo6gok6z13-wpengine.netdna-ssl.com/wp-content/uploads/2021/08/Castellanos-order-082021.pdf.

^21^Business Roundtable. “Stumbling on STEM: Why K-12 Education Must Align with the Digital Economy. Available online at: https://www.businessroundtable.org/stumbling-on-stem-why-k-12-education-must-align-with-the-digital-economy.

^22^Pwc. The 2018 digital university: Staying relevant in the digital age. Available online at: https://www.pwc.co.uk/assets/pdf/the-2018-digital-university-staying-relevant-in-the-digital-age.pdf.

## References

[B1] Alderman L. (2019). “The middle class Shrinks in Europe,” in The New York Times, February 16, at B1.

[B2] Alenezi I. (2021). Deep dive into digital transformation in higher education institutions. Educ. Sci. 11, 770

[B3] Anakpo G. Oyenubi A. (2022). Technological innovation and economic growth in Southern Africa: application of panel dynamic OLS regression. Dev. South. Afr. (2022) 39, 543–557. 10.1080/0376835X.2022.2052017

[B4] Andres K. B. Shaw T. (2020). Americans Face Financial Insecurity as Pandemic Hits. Aspen Institute. Available online at: https://www.aspeninstitute.org/blog-posts/americans-face-financial-insecurity-as-pandemic-hits/ (accessed July 1, 2022).

[B5] Arewa O. (2018). Investment funds, inequality, and scarcity of opportunity. Boston Univ. Law Rev. (2018) 99, 1023–1055.

[B6] Arewa O. (2021). Disrupting Africa: Technology, Law, and Development. New York, NY: Cambridge University Press, 175.

[B7] Ballotpedia (2022). California Proposition 22, App-Based Drivers as Contractors and Labor Policies Initiative. Available online at: https://ballotpedia.org/California_Proposition_22,_App-Based_Drivers_as_Contractors_and_Labor_Policies_Initiative_(2020) (accessed July 1, 2022).

[B8] Biron B. (2021). Amazon Workers in 20 Countries Will Strike or Protest on Black Friday for Better Working Conditions. BusinessInsider.com. Available online at: https://www.businessinsider.com/make-amazon-pay-campaign-staffers-will-strike-on-black-friday-2021-11 (accessed July 1, 2022).

[B9] Bivens J. Banerjee A. (2022). Inequality's Drag on Aggregate Demand. Economic Policy Institute Report. Available online at: https://files.epi.org/uploads/248892.pdf (accessed July 1, 2022).

[B10] Boesen M. Pedersen S. N. (2022). Proposal of EU-Directive on Improving Working Conditions in Platform Work Sets Out New Requirements to Digital Platforms. Bird & Bird. Available online at: https://www.twobirds.com/en/insights/2022/denmark/forslag-til-eu-direktiv-om-forbedring-af-arbejdsvilkaar (accessed July 1, 2022).

[B11] Bourguignon F. (2022). “Digitization and inequality,” in Shifting Paradigms: Growth, Finance, Jobs, and Inequality in the Digital Economy (Washington, DC: Brookings), 177–215.

[B12] Branson A. (2020). Empty Homes, Full Of promise – Part 2. RICS.org. Available online at: https://www.rics.org/de/wbef/megatrends/markets-geopolitics/empty-homes-full-of-promise–part-2/ (accessed July 1, 2022).

[B13] Brown H. C. (2018). Amazon Gets Huge Subsidies to Provide Good Jobs—but It's a Top Employer of SNAP Recipients in at Least Five States. The Counter. Available online at: http://thecounterorg.wpengine.com/amazon-snap-employees-five-states/ (accessed July 1, 2022).

[B14] Browning I. (2022). The Next Battleground for Gig Worker Labor Laws: Massachusetts. New York Times. Available online at: https://www.nytimes.com/2022/06/01/business/massachusetts-gig-workers-ballot.html (accessed July 1, 2022).

[B15] Brumer-Smith L. (2022). Are These 3 Things to Blame for Today's Housing Shortage?. MotleyFool.com. Available online at: https://www.fool.com/real-estate/2022/05/21/are-these-3-things-to-blame-for-todays-housing-sho/ (accessed July 1, 2022).

[B16] Brynjolfsson E. McAfee A. (2014). The Second Machine Age: Work, Progress, and Prosperity in a Time of Brilliant Technologies (New York, NY: Norton), at xiii.

[B17] Brynjolfsson E. Petropoulos G. (2021). Opinion: The Coming Productivity Boom. Cambridge, MA: MIT Technology Review.

[B18] Busby J. Tanberk J. (2020). FCC Reports Broadband Unavailable to 21.3 Million Americans, BroadbandNow Study Indicates that 42 Million Do Not Have Access. Available online at: https://broadbandnow.com/research/fcc-underestimates-unserved-by-50-percent (accessed July 1, 2022).

[B19] Cherewka A. (2020). The Digital Divide Hits U.S. Immigrant Households Disproportionately During the COVID-19 Pandemic. Migration Policy Institute. Available online at: https://www.migrationpolicy.org/article/digital-divide-hits-us-immigrant-households-during-covid-19 (accessed July 1, 2022).

[B20] Cohen J. (2007). Cyberspace as/and space. Columbia Law Rev. 107, 210–256, at 242.

[B21] Coppola D. (2022). Number of Amazon.com employees 2007-2021. Statistica.com. Available online at: https://www.statista.com/statistics/234488/number-of-amazon-employees/ (accessed July 1, 2022).

[B22] Cox M. Haar K. (2020). Platform Failures: How Short-Term Rental Platforms Like Airbnb Fail to Cooperate With Cities and the Need for Strong Regulations to Protect Housing, Study Commissioned by Members of the IMCO Committee of the GUE/NGL Group in the European Parliament. Available online at: http://insideairbnb.com/reports/platform-failures-how-short-term-rental-platforms-like-airbnb-fail-cities-en.pdf (accessed July 1, 2022).

[B23] Craig R. (2021). How Higher Ed Can Prepare Students for Today's Digital Jobs. Harvard Business Review. Available online at: https://hbr.org/2021/11/how-higher-ed-can-prepare-students-for-todays-digital-jobs (accessed July 1, 2022).

[B24] Dubal V. B. (2017). Wage slave or entrepreneur? Contesting the dualism of legal worker identities. Calif. Law Rev. 105, 65–123, at 72–80.

[B25] European Commission (2021). Proposal for a Directive of the European Parliament and of the Council on Improving Working Conditions in Platform Work. Available online at: https://ec.europa.eu/social/BlobServlet?docId=24992&langId=en (accessed July 1, 2022).

[B26] Federal Communications Commission (2019). Broadband Deployment Report. Available online at: https://docs.fcc.gov/public/attachments/FCC-19-44A1.pdf (accessed July 1, 2022).

[B27] Franz F. (2020). Home Office Could Be Here to Stay in Germany – if the Internet in Rural Areas Holds Up. Brussels: Heinrich Böll Stiftung. Available online at: https://eu.boell.org/en/2020/06/02/home-office-could-be-here-stay-germany-if-internet-rural-areas-holds (accessed July 1, 2022).

[B28] Frischmann B. M. Lemley M. A. (2007). Spillovers. Columbia Law Rev. 107, 257−301.

[B29] Giemzo J. Gu M. Kaplan J. Vinter L. (2020). How CIOs and CTOs Can Accelerate Digital Transformations Through Cloud Platforms. McKinsey Digital. Available online at: https://www.mckinsey.com/business-functions/mckinsey-digital/our-insights/how-cios-and-ctos-can-accelerate-digital-transformations-through-cloud-platforms (accessed July 1, 2022).

[B30] Goodwin h. (2020). Jeff Bezos Could Give All Amazon Workers $105,000 and Still be as Rich as Pre-Covid. The London Economic. Available online at: https://www.thelondoneconomic.com/news/jeff-bezos-could-give-all-amazon-workers-105000-and-still-be-as-rich-as-pre-covid-212545/ (accessed July 1, 2022).

[B31] Graves J. B. (2018). “The original gig economy, many futures of work,” in Possibilities and Perils 2018 Conference. Available online at: https://www.futuresofwork.org/s/Graves-Orig-Gig-Econ-II.pdf (accessed July 1, 2022).

[B32] Greene J. (2021). Amazon's Employee Surveillance Fuels Unionization Efforts: ‘It's Not Prison, It's Work'. Washington Post. Available online at: https://www.washingtonpost.com/technology/2021/12/02/amazon-workplace-monitoring-unions/ (accessed July 1, 2022).

[B33] Hendrickson C. Muro M. Galston W. A. (2018). Countering The Geography Of Discontent: Strategies For Left-Behind Places. Brookings Institution. Available online at: https://www.brookings.edu/research/countering-the-geography-of-discontent-strategies-for-left-behind-places/ (accessed July 1, 2022).

[B34] Henwood F. Wyatt S. (2019). Technology and in/equality, questioning the information society. Digi. Cult. Soc. 5, 183–194. Available online at: http://digicults.org/files/2020/12/11.-DCS_Inequalities_20-years-later.pdf (accessed July 1, 2022).

[B35] Herrera S. (2021). Amazon emerges as the wage-and-benefits setter for low-skilled workers across industries. *Wall Street J*. Available online at: https://www.wsj.com/articles/amazon-emerges-as-the-wage-and-benefits-setter-for-low-skilled-workers-across-industries-11638910694 (accessed July 1, 2022).

[B36] International Monetary Fund (2019). World Economic Outlook, 65. Available online at: https://www.imf.org/en/Publications/WEO/Issues/2019/10/01/world-economic-outlook-october-2019 (accessed July 1, 2022).

[B37] Jacobs K. Perry I. E. MacGillvary J. (2015). The High Public Cost of Low Wages. U.C. Berkeley Center for Labor Research and Education, 2. Available online at: https://laborcenter.berkeley.edu/the-high-public-cost-of-low-wages/ (accessed July 1, 2022).

[B38] Johnson D. G. (2006). “Introduction,” in Women, Gender and Technology, eds M. F. Fox, D. G. Johnson, and S. V. Rosser (Champaign: University of Illinois), 1.

[B39] Kantor J. Weise K. Ashford G. (2021). Inside Amazon's Worst Human Resources Problem. New York Times. Available online at: https://www.nytimes.com/2021/10/24/technology/amazon-employee-leave-errors.html (accessed July 1, 2022).

[B40] Kenney M. von Burg U. (1999). Technology, “Entrepreneurship and path dependence: industrial clustering in silicon valley and route 128. Ind. Corp. Change 8, 67–103, at 67.

[B41] Kimmelman M. (2018). France's Yellow Vests Reveal a Crisis of Mobility in All Its Forms. The New York Times, December 21, at A4.

[B42] Klippenstein K. (2022). Leaked: New Amazon Worker Chat App Would Ban Words Like ‘Union,' ‘Restrooms,' ‘Pay Raise,' And ‘Plantation'. The Intercept. Available online at: https://theintercept.com/2022/04/04/amazon-union-living-wage-restrooms-chat-app/ (accessed July 1, 2022).

[B43] Komarčević M. Dimić M. Celik P. (2017). Challenges and impacts of the digital transformation of society in the social sphere. SEER 20, 31–48.

[B44] Leggon A. B. (2006). “Gender, race/ethnicity, and the digital divide,” in Women, Gender and Technology, eds M. F. Fox, D. G. Johnson, and S. V. Rosser (Champaign, IL: University of Illinois), 98.

[B45] Leiserson G. Yagan D. (2021). What Is the Average Federal Individual Income Tax Rate on the Wealthiest Americans? The White House Blog. Available online at: https://www.whitehouse.gov/cea/written-materials/2021/09/23/what-is-the-average-federal-individual-income-tax-rate-on-the-wealthiest-americans/ (accessed July 1, 2022).

[B46] Leon L. F. (2021). A Worldwide Workers' Revolt Against Amazon Has Begun. In These Times. Available online at: https://inthesetimes.com/article/workers-world-unite-amazon-union-busting-organizing-labor-rights (accessed July 1, 2022).

[B47] Li H. Kim Y. Srinivasan K. (2021). Market Shifts in the Sharing Economy: The Impact of Airbnb on Housing Rentals, Working Paper. Available online at: https://ssrn.com/abstract=3435105 (accessed July 1, 2022).

[B48] Martineau P. (2019). Inside Airbnb's ‘Guerrilla War' Against Local Governments. Wired. Available online at: https://www.wired.com/story/inside-airbnbs-guerrilla-war-against-local-governments/ (accessed July 1, 2022).

[B49] Miller R. (2016). How AWS Came to Be, TechCrunch.com. Available online at: https://techcrunch.com/2016/07/02/andy-jassys-brief-history-of-the-genesis-of-aws/ (accessed July 1, 2022).

[B50] Mollaneda I. (2021). The Aftermath of California's Proposition 22. California Law Review Blog.

[B51] Morath E. Francis T. Baer J. (2020). The Covid economy carves deep divide between haves and have-nots. *Wall Street J*. Available online at: https://www.wsj.com/articles/the-covid-economy-carves-deep-divide-between-haves-and-have-nots-11601910595 (accessed July 1, 2022).

[B52] Newkirk II V. R. (2019). The Shutdown Showed How Precarious Americans' Finances Really Are. Atlantic. Available online at: https://www.theatlantic.com/politics/archive/2019/01/shutdown-laid-bare-americas-financial-precarity/581539/ (accessed July 1, 2022).

[B53] Nieuwland S. van Melik R. (2020). Regulating Airbnb: how cities deal with perceived negative externalities of short-term rentals. Curr. Issues Tour. 23, 811–825. 10.1080/13683500.2018.1504899

[B54] Oxfam America (2022). Tax the Rich, Build a Better World. Available online at: https://webassets.oxfamamerica.org/media/documents/Tax_the_rich_2022.pdf?_gl=1*1ka3932*_ga*MTAwNjA4NTU4OS4xNjU0NTQ0OTc2*_ga_R58YETD6XK*MTY1NDU0NDk3Ni4xLjEuMTY1NDU0NjE4Ny42MA (accessed July 1, 2022).

[B55] Parmeter K. C. (2016). From Amazon to Uber: defining employment in the modern economy. Boston Univ. Law Rev. 96, 1673–1728, at 1674–1675.

[B56] Piketty T. (2017). Capital in the Twenty-First Century. Cambridge: Harvard University Press, 405.

[B57] Putzier K. (2022). That vacation home listed on Airbnb might be owned by wall street. *Wall Street J*. Available online at: https://www.wsj.com/articles/that-vacation-home-listed-on-airbnb-might-be-owned-by-wall-street-11644930000 (accessed July 1, 2022).

[B58] Qureshi A. Woo C. (eds.) (2022). “Overview: digital metamorphosis and economic change,” in Shifting Paradigms: Growth, Finance, Jobs, and Inequality in the Digital Economy (Washington, DC: Brookings), 1–34.

[B59] Resca A. Spagnoletti P. (2014). “Business development through digital transformation: the evolution of Amazon.com,” in Information Systems, Management Organization and Control: Smart Practices and Effects, eds D. Baglieri, C. Metallo, C. Rossignoli, and M. P. Iacono (Heidelberg: Springer), 163–177.

[B60] Romer P. M. (1990). Endogenous technological change. J. Polit. Econ. 98, S71–S102, at S272

[B61] Rotman D. (2014). Technology and Inequality. MIT Technology Review. Available online at: https://www.technologyreview.com/2014/10/21/170679/technology-and-inequality/ (accessed July 1, 2022).

[B62] Sainato M. (2020). ‘*I'm not a robot': Amazon Workers Condemn Unsafe, Grueling Conditions at Warehouse*. Guardian. Available online at: https://www.theguardian.com/technology/2020/feb/05/amazon-workers-protest-unsafe-grueling-conditions-warehouse (accessed July 1, 2022).

[B63] Satariano A. Peltier E. (2021). Europe Pushes New Rules Turning Gig Workers Into Employees. New York Times. Available online at: https://www.nytimes.com/2021/12/09/technology/european-commission-gig-workers-uber.html (accessed July 1, 2022).

[B64] Saxena P. (2021). Amazon: Are Worker-Related Issues a Tipping Point? Newton Investment Management. Available online at: https://www.newtonim.com/uk-lgps/insights/articles/amazon-are-worker-related-issues-at-a-tipping-point/ (accessed July 1, 2022).

[B65] Schwab K. (2016). The Fourth Industrial Revolution: What it Means, How to Respond. World Economic Forum. Available online at: https://www.weforum.org/agenda/2016/01/the-fourth-industrial-revolution-what-it-means-and-how-to-respond/ (accessed July 1, 2022).

[B66] Schwertner K. (2021). “The impact of digital transformation on business: a detailed review,” in Strategic Management in the Age of Digital Transformation, ed J. Metselaar (London: Proud Penn), 1–29.

[B67] Segal E. (2021). Amazon's Next Crisis—Global Protests And Strikes on Black Friday. Forbes. Available online at: https://www.forbes.com/sites/edwardsegal/2021/11/25/amazon-prepares-for-next-crisis—a-global-strike-by-workers-on-black-friday/ (accessed July 1, 2022).

[B68] Semuels A. (2016). The Decline of Social Mobility in the United States. Atlantic Magazine. Available online at: https://www.theatlantic.com/business/archive/2016/07/social-mobility-america/491240/ (accessed July 1, 2022).

[B69] Sen A. (2000). Development as Freedom. New York, NY: Anchor).

[B70] Shead S. (2020). Ride-Hailing App Ola Stripped of London License Over Safety Concerns, Shortly After Uber Wins Reprieve. CNBC.com. Available online at: https://www.cnbc.com/2020/10/05/ola-ride-hailing-app-banned-in-london-by-tfl-over-safety-concerns.html (accessed July 1, 2022).

[B71] Siripurapu A. (2022). Backgrounder: The U.S. Inequality Debate, Council on Foreign Relations. Available online at: https://www.cfr.org/backgrounder/us-inequality-debate (accessed July 1, 2022).

[B72] Statistica Research Department (2022). Global Digital Population as of April 2022. Statistica.com. Available online at: https://www.statista.com/statistics/617136/digital-population-worldwide/ (accessed July 1, 2022).

[B73] Streitfeld D. (2018). Was Amazon's Headquarters Contest a Bait-and-Switch? Critics Say Yes. New York Times. Available online at: https://www.nytimes.com/2018/11/06/technology/amazon-hq2-long-island-city-virginia.html (accessed July 1, 2022).

[B74] Tartar A. Pickert R. (2019). A Third of America's Economy Is Concentrated in Just 31 Counties. Bloomberg.com. Available online at: https://www.bloomberg.com/graphics/2019-us-gdp-concentration-counties/ (accessed July 1, 2022).

[B75] Thomas K. P. Tarczynska K. Martinez A. Wen C. LeRoy G. (2022). Amazon.com's Hidden Worldwide Subsidies, UNI Global Union and Good Jobs First Report. Available online at: https://uniglobalunion.org/wp-content/uploads/amazon_subsidies_final.pdf (accessed July 1, 2022).

[B76] Torpey E. Hogan A. (2016). Working in a Gig Economy. U.S. Bureau of Labor Statistics. Available online at: https://www.bls.gov/careeroutlook/2016/article/pdf/what-is-the-gig-economy.pdf (accessed July 1, 2022).

[B77] Tramontano K. a. (2017). America Is Failing Workers Left Behind by the New Economy. Washington Monthly. Available online at: https://washingtonmonthly.com/2017/03/07/america-is-failing-workers-left-behind-by-the-new-economy/, https://perma.c~c/2NAT-CF44 (accessed July 1, 2022).

[B78] United Nations (2020). Digital Divide ‘a Matter of Life and Death' amid COVID-19 Crisis, Secretary-General Warns Virtual Meeting, Stressing Universal Connectivity Key for Health, Development. Available online at: https://www.un.org/press/en/2020/sgsm20118.doc.htm (accessed July 1, 2022).

[B79] Weise K. Scheiber N. (2022). Amazon Workers on Staten Island Vote to Unionize in Landmark Win for Labor. New York Times. Available online at: https://www.nytimes.com/2022/04/01/technology/amazon-union-staten-island.html (accessed July 1, 2022).

[B80] Wilhelm A. G. (2004). Digital Nation: Towards an Inclusive Information Society. Cambridge: MIT Press.

[B81] Williams A. (2021). 5 Ways Amazon Monitors Its Employees, From AI Cameras to Hiring a Spy Agency. BusinessInsider.com. Available online at: https://www.businessinsider.com/how-amazon-monitors-employees-ai-cameras-union-surveillance-spy-agency-2021-4 (accessed July 1, 2022).

[B82] Wilson F. (2019). Capitalism and Inequality. AVC Blog. Available online at: https://avc.com/2019/01/capitalism-and-inequality/, https://perma.cc/43CW-R5JX (accessed July 1, 2022).

[B83] World Economic Forum (2016). What is the Fourth Industrial Revolution? Available online at: https://www.weforum.org/agenda/2016/01/what-is-the-fourth-industrial-revolution/ (accessed July 1, 2022).

[B84] Wuthnow R. (2018). The Left Behind: Decline and Rage in Rural America. Princeton: Princeton University Press, 1–4.

[B85] Wyatt A. Henwood F. Miller N. Senker P. (eds.). (2000). Technology and In/equality: Questioning the Information Society. New York, NY: Routledge.

[B86] Yglesias M. (2017). Uber's Toxic Culture of Rule Breaking, Explained. Vox.com. Available online at: https://www.vox.com/new-money/2017/3/21/14980502/uber-toxic-culture-rule-breaking-explained (accessed July 1, 2022).

[B87] Zaimes I. C. Kreeger N. C. (2021). California Judge Enjoins Proposition 22 as Unconstitutional in Classifying App-Based Drivers as Independent Contractors, Arent Fox Perspectives. Available online at: https://www.arentfox.com/perspectives/alerts/california-judge-enjoins-proposition-22-unconstitutional-classifying-app-based (accessed July 1, 2022).

